# Clear cell tumor of the lung: a case report and literature review

**DOI:** 10.1186/1477-7819-11-247

**Published:** 2013-10-01

**Authors:** Guang-xian Wang, Dong Zhang, Xin-wei Diao, Li Wen

**Affiliations:** 1 Department of Radiology, Xinqiao Hospital, Third Military Medical University, Chongqing 400037, China; 2 Department of Pathology, Xinqiao Hospital, Third Military Medical University, Chongqing 400037, China

**Keywords:** Clear cell tumor, Sugar tumor, Lung

## Abstract

Clear cell tumor of the lung is a rare and benign pulmonary tumor; only sporadic cases have been reported. Here, we report the case of a 38-year-old man with recurrent cough, blood-streaked sputum and left chest pain. A chest computed tomography scan showed a round, homogeneous pulmonary mass in the left lower lobe, which exhibited intense heterogeneous enhancement in the arterial phase and homogeneous in the delay phase after injecting a contrast agent. The patient underwent a fine-needle aspiration biopsy and was diagnosed as having a benign clear cell tumor of the lung. The clinical presentation and radiographic investigation of this tumor are summarized in this paper to recognize this rare disease. Interestingly, we found some differences with previously reported cases.

## Background

Clear cell tumor of the lung (CCTL) is a rare and benign pulmonary neoplasm, which was originally described by Liebow and Castleman in 1963 [1]. CCTL most likely arises from perivascular epithelioid cells (PECs) [2-4]. As it contains abundant cytoplasmic periodic acid-Schiff (PAS)-positive glycogen, CCTL has been termed “sugar tumor” [5]. The tumor cells show immunoreactivity for the S-100 protein and HMB-45 and no cytokeratin reactivity, which establish a definitive diagnosis [6]. Although the tumor has been well defined, only sporadic cases have been reported in the literature. Here, we report on a CCTL case and summarize a literature review of 55 CCTL cases. Our aims were to identify the clinical and radiological features of CCTL and determine the effect of CCTL tumor size.

## Case presentation

**Figure 1:**
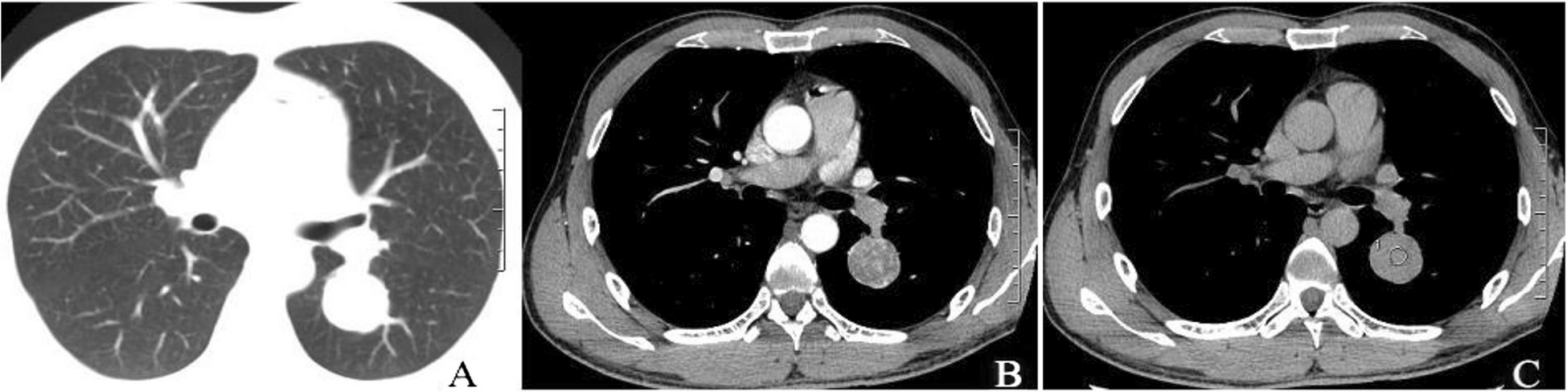
**Chest computed tomography findings of the tumor. (A)** Chest plain computed tomography scan shows a solitary, rounded, smooth-surfaced, homogenous density mass of approximately 3.4 cm in diameter in the left lower lobe (S6). **(B)** The contrast-enhanced arterial phase shows intense heterogeneous enhancement measuring 67.2 to 128.0 Hounsfield units. **(C)** The contrast-enhanced delay phase shows an isodense nodule measuring 66.3 Hounsfield units.

A 38-year-old man was admitted to our hospital because of recurrent cough, blood-streaked sputum for 2 months, left chest pain for 10 days, and worsening health for 3 days. The patient, who had no known family history of cancer, was an 18 pack/year smoker. He did not report any fever, night sweating, chest tightness, weight loss, or wheezing. A physical examination did not show any abnormalities, and the results of laboratory studies were unremarkable. Chest non-enhanced computed tomography (CT) scans showed a demarcated, round, homogeneous lesion of approximately 3.4 cm in diameter without evidence of calcification, necrosis, cavitation, or satellite lesions in the left lower lobe (Figure 1A), with a CT value of 46.6 Hounsfield units. A contrast-enhanced CT scan of the solitary pulmonary mass showed intense heterogeneous enhancement in the arterial phase (Figure 1B) and a homogeneous nature in the delay phase (delay 150 seconds) (Figure 1C). Abdominal CT scans showed no evidence of renal disease.

Using an 18-gauge needle, a CT-guided biopsy of the lung at our institution was performed to determine the nature of the lesion. Light microscopy and immunohistochemical studies were performed. The material for pathological examination that was obtained at the biopsy was fixed in 10% formalin and embedded in paraffin. For light microscopy, 5-μm thick sections were cut from the inclusions and stained with hematoxylin and eosin and PAS. For PAS staining, the slide was fixed with 4% paraformaldehyde, oxidized in 1% periodic acid for 5 minutes and then washed and treated with Schiff’s reagent for 15 minutes. The slide was subsequently treated for color development in dH_2_O for 5 to 10 minutes and assessed under a light microscope. For immunohistochemistry, paraffin sections were treated with monoclonal antibodies against HMB-45, vimentin, CD34, S-100 protein, cytokeratin, desmin, CD68, EMA, RCC, and TTF-1. The antigen-antibody complexes were visualized using the streptavidin-biotin-peroxidase complex method.

**Figure 2:**
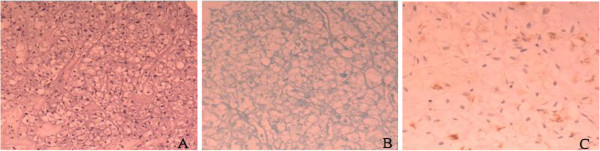
**Microscopic and immunohistochemistry findings. (A)** Histopathological examination shows diffuse growth of rounded or oval cells with abundant clear cytoplasm, distinctive cell borders, and thin-walled blood vessels (hematoxylin and eosin, ×200. **(B)** Most neoplastic clear cells have intracytoplasmic periodic acid-Schiff-positive glycogen granules (streptavidin-perosidase, ×200. **(C)** Many tumor cells show cytoplasmic staining for HMB-45 (streptavidin-perosidase, ×200).

Histologically, the lesion consisted of sheets of neoplastic cells. Plump, short, polygonal, and spindle-shaped cells with distinctive cell borders and mildly pleiomorphic nuclei were observed. These cells were surrounded by thin-walled blood vessels of various sizes (Figure 2A). No signs of necrosis and no mitotic figures were seen. The clear cytoplasm contained numerous glycogen granules, as demonstrated by PAS stain (Figure 2B). The immunohistochemical studies showed strong immunoreactivity for HMB-45 (Figure 2C) and showed a positive reaction to vimentin, CD34, and S-100 protein but no reactivity for cytokeratin, desmin, CD68, EMA, RCC, and TTF-1. Based on these findings, the lesion was diagnosed as CCTL. The patient underwent a left thoracotomy, and a wedge resection was performed to remove the tumor, whose pathologic findings were concordant with the biopsy specimen. No evidence of recurrence or metastatic disease was evident during the 12-month follow-up period after the surgery.

### Literature review

PubMed and Google were searched in March 2012 using the following terms in various combinations: benign lung tumor, clear cell tumor, sugar tumor, CCTL and PEComa. Only full-text English studies were reviewed.

A total of 56 CCTL cases (including the present case) were reviewed [2,4,6-27]. The collected data included patient age, gender, symptoms, and tumor location, contour, and density. Table 1 illustrates the clinical characteristics of the CCTL patients and radiological features of the tumors. The relationships between tumor size and the parameters of symptoms, location, contour, and density were assessed with Spearman’s correlation. The statistical differences in symptoms and tumor contour were compared between the ≥2.2 cm tumor size group and the <2.2 cm tumor size group using the chi-squared test. Statistical analyses were performed with the Statistical Package for the Social Sciences (SPSS, Chicago IL, U.S.A; version 17.0). A *P* value <0.05 was considered statistically significant.

**Table 1:** **Composite presentation of 56 CCTL cases**[2,4,6-27]

	***n *** **(%)**
Age (years)	
<40	12/56 (21.4)
40-70	41/56 (73.2)
>70	3/56 (5.4)
Sex	
Male	29/56 (51.8)
Female	27/56 (48.2)
Symptoms	
Asymptomatic	37/56 (66.1)
Symptomatic	19/56 (33.9)
Location	
LU	9/52 (17.3)
LL	19/52 (36.5)
RU	8/52 (15.4)
RM	3/52 (5.8)
RL	13/52 (25.0)
NR	4
Size (cm)	
<2.2	29/56 (51.8)
≥2.2	27/56 (48.2)
Contour	
Round	39/44 (88.6)
Lobulated	5/44 (11.4)
NR	12
Density	
Homogeneous	42/51 (82.4)
Heterogeneous	9/51 (17.6)
NR	5

LU, left upper lobe; LL, left lower lobe; RU, right upper lobe; RM, right middle lobe; RL, right lower lobe; NR, not recorded.

Spearman’s correlation analysis showed that tumor size was most frequently associated with symptoms and tumor contours (Table 2), whereas it has no significant correlation with location and density (Table 2). There were significant statistical differences in patient symptoms and tumor contour between the ≥2.2 cm group and the <2.2 cm group. Patients were more likely to experience symptoms (chi^2^ = 56.000, *P* = 0.000, Table 3), and the tumor was more likely to be lobulated (chi^2^ = 72.333, *P* = 0.000, Table 4) if the tumor size was ≥2.2 cm.

**Table 2:** Correlation between tumor size and symptoms, location, contour, and density

	**Correlation coefficient (r**_**s**_**)**	***P***
Symptom	0.475	0.000
Location	-0.041	0.763
Contour	0.344	0.01
Density	0.052	0.702

**Table 3:** Correlation between tumor size and symptoms

**Size**	Asymptomatic*	**Symptomatic**	**Total**
<2.2 cm	25	4	29
≥2.2 cm	12	15	27

*Four cases with symptoms that were not necessarily connected with CCTL were classified as asymptomatic.

**Table 4:** Correlation between tumor size and contour

**Size**	**Round**	**Lobulated**	**NR**	**Total**
<2.2 cm	24	0	5	29
≥2.2 cm	15	5	7	27

## Discussion

CCTL is a rare benign pulmonary neoplasm originally described in 1963 by Liebow and Castleman [1]. Since that time, fewer than 60 cases have been reported in the English literature. CCTL can occur in any age group (range 8 to 73 years), with equal sex prevalence [7,8,10,16,17,23,25,26] or a slight female predominance [4,9,14,24,28]. However, in our study, males were slightly more prone to CCTL than females (29/27). Most patients were asymptomatic; only a few patients displayed symptoms (for example, chest pain, back pain, breathlessness, sense of suppression, cough, pneumonia, fever, bloody sputum or hemoptysis) [6-15,24]. These symptoms were usually nonspecific, as most cases were discovered incidentally on routine chest radiographs or CT scans.

Radiographically, CCTL presents as a rounded, smooth-walled peripheral parenchymal nodule, without evidence of cavitation or calcification. There are no specific lobar distributions according to the literature [7,8,16,17]. In our study, nodules were generally found in the lower lobes of both lungs (32/52) and were unrelated to vessels or major bronchi. The tumor sizes ranged from 1 mm [29] to 12 cm [10]. In our literature review, we found that certain clinicopathologic features such as a diameter >2.5 cm, the presence of symptoms, and extensive necrosis or abundant mitoses visible under an optical microscope were associated with more aggressive behavior [13,17,21,27]. Our results showed that the tumor size was most closely correlated with patients’ symptoms and tumor contour: significant statistical differences were found between the ≥2.2 cm group and the <2.2 cm group. The results suggested that patients should undergo operation or follow-up if the tumor size was ≥2.2 cm. However, the lesion density was not correlated with tumor size.

Based on a contrast-enhanced CT scan, the main features of this case were the intense heterogeneous enhancement in the arterial phase and washout in the delay phase, which were consistent with a previous report [17]. This CT finding appears to be a result of the vascular stroma [8,13,17,24], although occasionally, for an unknown reason, there is no enhancement [22]. The characteristics of CCTL, which have been described as having an intense heterogeneous enhancement in the arterial phase and homogeneous in the delay phase, have not been previously reported. The CT enhancement features indicate the malignant potential of this mass rather than a benign nodule [17]. The preoperative accurate CT diagnosis of this disease is difficult.

CCTL is usually diagnosed by thoracotomy, lobectomy, and segmentectomy. Partial resection and enucleation have been reported as treatments in previous studies [22]. Only one case of CCTL was diagnosed pre-operatively by a transbronchial lung biopsy [19], and three cases (including the case reported here) by fine-needle aspiration biopsy [9,23]. The biology of this tumor has traditionally been considered benign, but malignant behavior has been occasionally reported. A 1988 report described a patient with CCTL who died from metastatic CCTL [30], and two other reports described this tumor with rapid growth [5,26]. Our patient was a heavy smoker with a lung mass, which exhibited an intense post-contrast enhancement in the arterial phase and washout in the delay phase on CT scan and was considered to have a malignant potential. Surgical treatment was suggested.

Macroscopically, CCTL predominantly appears as well-circumscribed, peripheral nodules measuring ≤3 cm in diameter; cut surfaces are typically homogeneous and glistening, without evidence of hemorrhage, necrosis, cavitation, or calcification [8,17]. Histologically, the nodules are composed of mitotically inactive round or oval cells with clear or granular eosinophilic cytoplasm and distinct cell borders, with characteristic intervening thin-walled sinusoidal vessels; spindle cells may be observed occasionally [9]. PAS stain shows glycogen granules in the cytoplasm of these clear cells, which is why they are referred to as sugar tumors [5]. The clear cell tumor has to be differentiated from other malignant clear-cell tumors, particularly unusual primary clear cell adenocarcinoma of the lung and metastatic renal cell carcinoma, both of which can have a bland morphologic appearance and, therefore, can mimic CCTL. The tumor cells in both primary and secondary clear cell adenocarcinomas of the lung are positive for cytokeratin, whereas CCTL cells are always non-reactive for cytokeratin [22]. In our case, the diagnosis was based on the typical histology picture of the tumor, the positivity for PAS and HMB-45, and the absence of clinical signs of a renal tumor. We believe that the joint application of these markers can distinguish CCTL from other pulmonary neoplasms with greater precision [25].

## Conclusions

In summary, we reported a case of CCTL and a literature review of 55 CCTL cases. We analyzed the patient’s clinical characteristics, the tumor’s radiological features, and the effects of CCTL tumor size. We conclude that the tumor has three characteristics: (1) most of the patients are middle-aged and elderly people (with slightly more males than females) and are generally asymptomatic; (2) the tumor, which is usually located in both lower lungs, is round and homogeneous density on plain CT scan image; and (3) tumors ≥2.2 cm in diameter grow lobulated and have more aggressive symptoms.

## Consent

Written informed consent was obtained from the patient for publication of this case report and the accompanying images. Copies of the written consent are available for review upon request.

## Abbreviations

CCTL: Clear cell tumor of the lung; CT: Computed tomography; PAS: Periodic acid-Schiff; PEC: Perivascular epithelioid cells.

## Competing interests

The authors declare that they have no competing interests.

## Authors’ contributions

GW performed the research. LW designed the research study. XD performed the pathological examination. GW and DZ analyzed the data. All authors read and approved the final manuscript.
